# Unilateral Urrets-Zavalia syndrome after Implantable Collamer Lens implantation: a case report and review of the literature

**DOI:** 10.1186/s13256-021-03063-2

**Published:** 2021-09-22

**Authors:** Duangratn Niruthisard, Ngamjit Kasetsuwan

**Affiliations:** Department of Ophthalmology, Faculty of Medicine, Chulalongkorn University, 1873 Rama 4 road, Pathumwan, Bangkok, 10330 Thailand

**Keywords:** Urrets-Zavalia syndrome, Implantable Collamer Lens implantation, Phakic intraocular lens implantation

## Abstract

**Background:**

Fixed dilated pupil after ophthalmic surgery or Urrets-Zavalia syndrome occurs after anterior segment surgery and usually relates to postoperative elevation of intraocular pressure. Urrets-Zavalia syndrome results in complaints of glare, halo, and photophobia. Retention of the viscoelastic agent during Implantable Collamer Lens implantation can result in postoperative elevation of intraocular pressure and Urrets-Zavalia syndrome. However, reversibility of pupillary dilatation is possible in some cases.

**Case presentation:**

A 20-year-old Thai man with myopic astigmatism in both eyes underwent Implantable Collamer Lens implantation in the right eye. The preoperative slit-lamp examination of both eyes was normal, and no ectatic changes were detected from corneal tomography. One hour after the uncomplicated surgery of the right eye, intraocular pressure increased to 48 mmHg and was immediately controlled with antiglaucoma medications. Postoperative pupillary dilatation was detected, presumably due to effect of preoperative application of mydriatic drops. At postoperative day 1, the right pupil remained dilated but still reactive to light and pilocarpine 2% eye drops. Two weeks later, the left eye underwent the Implantable Collamer Lens implantation and showed neither postoperative increase in intraocular pressure nor postoperative pupillary dilatation. Two months after surgery, the dilatation of the right pupil partially reversed.

**Conclusions:**

The findings of the right eye suggested diagnosis of Urrets-Zavalia syndrome. Compared with former reports, we noted an association between immediate control of elevation of postoperative intraocular pressure, light reactivity of the dilated pupil, and reactivity to pilocarpine 2% eye drops as potential predictors for reversibility of Urrets-Zavalia syndrome.

## Background

Fixed dilated pupil after ophthalmic surgery or Urrets-Zavalia syndrome (UZS) was first described following penetrating keratoplasty in keratoconus [1], and an association with postoperative elevation of intraocular pressure (IOP) was reported [2]. The majority of UZS cases occurred after penetrating keratoplasty in keratoconus [3, 4]. However, UZS was also reported to follow other anterior segment ophthalmic surgeries such as deep anterior lamellar keratoplasty (DALK) [5], Descemet’s membrane stripping automated endothelial keratoplasty (DSAEK) [6], glaucoma surgeries [7–9], cataract surgery [10], and phakic intraocular lens implantation [11–16].

We reported a case of UZS following Implantable Collamer Lens (ICL) implantation. By reviewing previous literature, we attempted to identify possible risk factors of this syndrome and factors indicating reversibility of the dilated pupil.

## Case presentation

A 20-year-old healthy Thai man with myopic astigmatism attended our clinic for refractive surgery. He denied any past ophthalmic procedures. Best-corrected visual acuity (BCVA) was 20/32 in the right eye (OD) with −7.25–3.75 × 15° and 20/40-1 in the left eye (OS) with −10.75–6.0 × 170°. Corneal tomography and corneal thickness were obtained with a rotating Scheimpflug camera system for anterior segment analysis (OCULUS Optikgeräte, Pentacam, GmbH Wetzlar, Germany). The result showed that the thickness of the thinnest point of the right and left cornea was 521 and 511 μm, respectively, and no ectatic changes were detected. Instead of undergoing laser vision correction, we recommended implantation of toric Implantable Collamer Lens (toric ICL; STAAR Surgical, Nidau, Switzerland) in both eyes owing to thin corneas. Two separate operations of ICL implantation were scheduled, starting with the right eye. Preoperatively, the slit-lamp examination was completely normal. His pupils were reactive to light, and the diameters of pupils were equal. His scotopic diameters of the right and left pupils measured with a corneal topography (Carl Zeiss, ATLAS^TM^ 9000 Corneal Topographer, Jena, Germany) using PathFinder II Corneal Analysis Software were 7.0 and 6.4 mm, respectively. The anterior chamber depth (ACD) measured with scanning slit corneal topography (Bausch & Lomb, Orbscan II, Rochester, NY, USA) was 3.32 mm in the right eye and 3.2 mm in the left eye. The white-to-white (WTW) distance measured with a caliper was 11.5 mm in both eyes. The preoperative intraocular pressures of the right and the left eyes were 17 and 16 mmHg, respectively. The patient did not require preoperative preventive iridotomies because of the presence of a hole at the center of the optic part of the fourth generation of ICL. The preoperative endothelial cell counts of the right and left eyes were 2728 and 2824 cells/mm^2^, respectively. The 12.6-mm ICLs were selected for both eyes. Before surgery, tropicamide 1% ophthalmic solution (Novartis, Mydriacyl 1%, UK) was applied until full pupillary dilatation was achieved. A viscoelastic agent (Alcon Laboratories, Inc., Provisc, Fort Worth, Texas, USA) was injected into the anterior chamber through a paracentesis port. The ICL was implanted in the right eye through a 2.75 mm temporal clear corneal incision. The viscoelastic agent was removed by manual irrigation with balance salt solution.

**Fig. 1:**
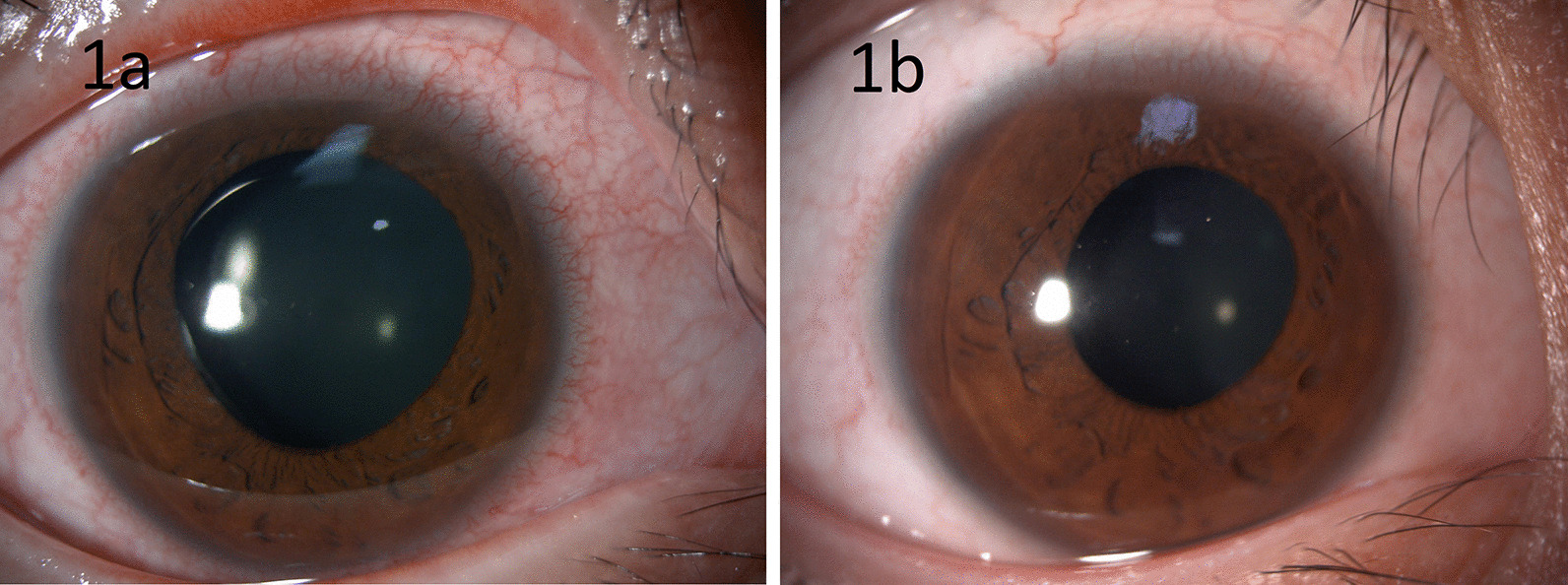
This picture shows pupillary dilatation of the right eye at postoperative 5 days (**a**). Reactivity to pilocarpine 2% eye drops resulted in partial reversibility of pupillary dilatation (**b**)

One hour after uncomplicated surgery, the patient complained of mild ocular pain. The IOP of the right eye measured with an applanation tonometer elevated to 48 mmHg. The cornea was clear, and the vault of ICL was 1.5 times of central corneal thickness (CCT). A 250 mg acetazolamide tablet was given orally, and the IOP decreased to 30 mmHg within 1 hour. The patient was then prescribed 250 mg acetazolamide tablets every 6 hours before he was discharged. On the first postoperative day, IOP decreased to 15 mmHg. Visual acuity was 20/30-2 and improved to 20/25-2 with pinhole correction. Refraction showed +2.5−1.75 × 60°. Although the IOP was normal and the cornea was clear, we saw retention of viscoelastic agent in the anterior chamber, and the right pupil was 6 mm in diameter and slightly reactive to light. Although the ICL was in place with a vault 1.75 times of CCT, we decided to remove the viscoelastic agent by irrigation with balance salt solution. On the next day, the visual acuity was 20/25-1 and improved to 20/20-1 with pinhole correction. Refraction showed +1.37−2.25 × 31°, and the IOP was 13 mmHg. The right pupil still dilated 6 mm in diameter. Five days after ICL implantation, the patient complained of glare especially at night despite satisfactory visual acuity. Despite normalized IOP, the right pupil remained 6 mm under slit-lamp examination (Fig. 1a) and was slightly reactive to light. Pilocarpine 2% eye drops (Alcon Laboratories, Inc., USA) were applied to the right eye every 15 minutes four times, and the pupil constricted to 3 mm. The patient had applied pilocarpine 2% eye drops in the morning for 2 consecutive days and complained of less glare. Therefore, we advised him to continue pilocarpine 2% eye drops once a day in the morning. The visual acuity was 20/25+2, the autorefraction was −1.00−2.50 × 45°, and IOP was 16 mmHg. The pupil was 4 mm in diameter and reactive to light (Fig. 1b). The ICL was in a good position with the 1.5-time CCT vault. We performed the ICL implantation in the left eye 2 weeks later. The surgery was successful without postoperative increased intraocular pressure, and his left pupil did not dilate after surgery.

**Fig. 2:**
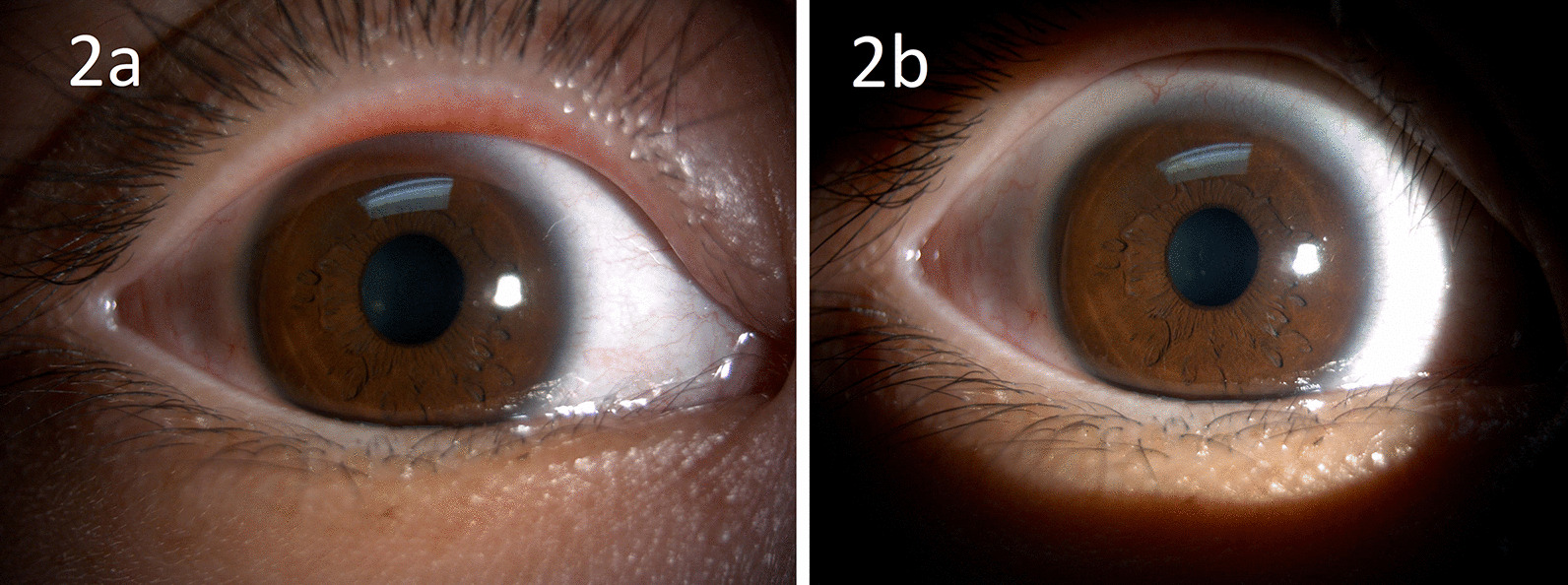
The right pupil (**a)** at postoperative 2 months after cessation of pilocarpine 2% eye drops for 1 month. The left pupil (**b)** after postoperative 2 months was also demonstrated for comparison

Two months after surgery, the patient stopped pilocarpine 2% eye drops owing to conjunctival injection. He visited our clinic 3 months after surgery and claimed that the night glare improved, although more glare was detected in the right eye compared with the left eye. However, no glare was experienced during the day. In general, he was satisfied with the results of surgery. On examination, his right eye was 20/20-2, while his left eye was 20/20. The  intraocular pressure was 13 mmHg in both eyes. The right pupil was 3.5 mm in diameter under slit-lamp examination and was reactive to light. The left pupil was 2.5 mm in diameter (Fig. 2). The higher-order aberrations in root mean square (RMS) of his right and left eyes measured by corneal topography (Carl Zeiss, ATLAS^TM^ 9000 Corneal Topographer, Jena, Germany) using PathFinder II Corneal Analysis Software were 0.37 and 0.43 µm, respectively. No area of iris atrophy was detected in the right eye.

## Discussion

Although the pathogenesis of UZS is controversial, there are two widely accepted mechanisms: iris ischemia and atrophy [3,^ 5^,^ 6^,^ 14^,^ 17^, 18] and injury to radial fibers of parasympathetic nerve [7]. Postoperative elevated IOP found in several anterior segment surgeries is a major cause of iris ischemia and atrophy. Tuft and colleagues reported UZS in three keratoconic patients who had elevation of IOP after penetrating keratoplasty and demonstrated delayed filling of iris vessels in isolated segments of the iris at 4–6 weeks postoperation by using anterior segment fluorescein angiography [2]. A summary of proposed causes of iris ischemia and atrophy can be found in Table 1.

**Table 1:** Proposed causes of iris ischemia and atrophy resulting in Urrets-Zavalia Syndrome (UZS)

●Increased intraocular pressure at postoperative period: Retention of a viscoelastic agent after phakic IOL implantation [14] Pupillary block due to air or gas bubble in the anterior chamber after anterior segment surgical procedures such as DALK, DSAEK, and intracameral C_3_F_8_ injection for treatment of acute corneal hydrops [5, 6, 17] ●Direct trauma to iris during penetrating keratoplasty [18] ●Vascular strangulation caused by intraocular contents pushing lens–iris diaphragm against the edge of host cornea [3, 12] ●Severe toxic anterior segment syndrome [19] ●Surgically induced hypromellose toxicity to vasculature or sphincter of iris [10]	

Literature review revealed 6 cases of phakic intraocular lens implantation demonstrating UZS as summarized in Table 2. Postoperative increase in IOP was found in five cases of UZS after phakic intraocular lens implantation. In most of these cases (4 out of 5 cases), the elevation of IOP was detected at postoperative day 1, not at the immediate postoperative period, and all of the 4 cases experienced irreversible fixed dilated pupils [11,^ 13^–15]. The intraocular pressure at postoperative day 1 ranged from 32 to 64 mmHg, and pupils were not reactive to pilocarpine 2% eye drops. In our case, the elevation of IOP was detected 1 hour after operation. The immediate vigorous treatment was initiated, and the IOP was normalized within 24 hours postoperation. The affected pupil was mid-dilated but still reactive to light and pilocarpine 2% eye drops. Three months after surgery, the pupillary dilatation partially reversed from 6 mm to 3.5 mm. Our case demonstrated the reversibility of pupillary dilatation similar to the previous case reported by Al-Thomali and Alabhar [16] in which the elevated IOP was normalized in the immediate postoperative period. Reactivity of the dilated pupils to light and pilocarpine 2% eye drops was also noted in both our case and the case reported by Al-Thomali and Alabhar [16].

**Table 2:** Summary of previous case reports of dilated pupils after phakic intraocular lens implantation

Author, year	Type of phakic IOL	Preexisting corneal disease	Increase in IOP		Cause of elevated IOP	Time when dilated pupil was detected	Characteristic of dilated pupil		Outcome at final follow-up	Treatment
			At immediate postoperative period	at postoperative day 1			Reactivity to light at early postoperative period	Reactivity to pilocarpine		
Anterior chamber phakic IOL implantation										
Yuzbasioglu *et al*. 2006 [11]	Iris-claw IOL	None	N/A	60 mmHg	N/A	1 day after surgery	No	No	Irreversible after postoperative 6 months	N/A
Park *et al*. 2008 [12]	Iris-claw IOL	None	N/A	No	N/A	2 weeks after surgery*	No	No	Irreversible after postoperative 2 months	N/A
Posterior chamber phakic IOL implantation										
Kummelil *et al*. 2011 [13]	ICL (STAAR Surgical, Nidau, Switzerland)	None	N/A	48 mmHg	Viscoelastic retention**	1 day after surgery	N/A	No	Irreversible after postoperative 3 months	Conservative
Perez-Cambrodi *et al*. 2014 [14]	Phakic refractive lens (PRL) (Carl Zeiss Meditec, Jena, Germany)	None	N/A	65 mmHg	Viscoelastic retention	1 day after surgery	Slightly reactive to light	No	Irreversible after postoperative 3 years	Tinted CL
Habash *et al*. 2015 [15]	ICL (STAAR Surgical, Nidau, Switzerland)	None	N/A	52 mmHg	N/A	1 day after surgery	No	No	Irreversible after postoperative 3 years	N/A
Al-Thomali *et al*. 2016 [16]	Toric ICL (STAAR Surgical, Nidau, Switzerland)	Nonprogressive keratoconus	1 hour after surgery: OD 26 mmHg OS 28 mmHg	IOP returned to normal at postoperative day 1	N/A	1 day after surgery	Yes	Yes	Partially reverse after postoperative 2 months	N/A
Our case	Toric ICL (STAAR Surgical, Nidau, Switzerland)	None	1 hour after surgery: OD 48 mmHg	IOP returned to normal at postoperative day 1	Viscoelastic retention	1 day after surgery	Yes	Yes	Partially reverse after postoperative 3 months	Conservative

CL, contact lens; ICL, implantable collamer lens; IOL, intraocular lens; IOP, intraocular pressure; N/A, not applicable

*Report of normal size pupil at postoperative day 1 and week 1

**The patient also had severe anterior uveitis in the early postoperative period

## Conclusions

In summary, we report a case of UZS in phakic IOL implantation. Compared with previous reports, the data showed that aggressive IOP control in the immediate postoperative period tends to provide a chance for reversibility of dilated pupils. We also noted that postoperative reactivity of dilated pupils to light and pilocarpine 2% eye drops may be indicators for reversibility of pupillary dilatation.

## Data Availability

All data and material collected during this study are available from the corresponding author upon reasonable request.

## References

[1] Urrets Zavalia A Jr. Fixed, dilated pupil, iris atrophy and secondary glaucoma. Am J Ophthalmol. 1963;56:257–65. 10.1016/0002-9394(63)91861-0 14061605

[2] Tuft SJ, Buckley RJ. Iris ischaemia following penetrating keratoplasty for keratoconus (Urrets-Zavalia syndrome). Cornea. 1995;14:618–22. 8575186

[3] Davies PD, Ruben M. The paretic pupil: its incidence and aetiology after keratoplasty for keratoconus. Br J Ophthalmol. 1975;59(4):223–8. 10.1136/bjo.59.4.223 PMC1042598 1095050

[4] Figueiredo GS, Kolli SS, Ahmad S, Gales K, Figueiredo FC. Urrets-Zavalia syndrome following penetrating keratoplasty for keratoconus. Graefes Arch Clin Exp Ophthalmol. 2013;251(3):809–15. 10.1007/s00417-012-2148-8 22965868

[5] Bozkurt KT, Acar BT, Acar S. Fixed dilated pupilla as a common complication of deep anterior lamellar keratoplasty complicated with Descemet membrane perforation. Eur J Ophthalmol. 2013;23(2):164–70. 10.5301/ejo.5000198 23065856

[6] Fournie P, Ponchel C, Malecaze F, Arne JL. Fixed dilated pupil (Urrets-Zavalia syndrome) and anterior subcapsular cataract formation after Descemet stripping endothelial keratoplasty. Cornea. 2009;28(10):1184–6. 10.1097/ICO.0b013e31819aaa13 19730095

[7] Espana EM, Ioannidis A, Tello C, Liebmann JM, Foster P, Ritch R. Urrets-Zavalia syndrome as a complication of argon laser peripheral iridoplasty. Br J Ophthalmol. 2007;91(4):427–9. 10.1136/bjo.2006.105098 PMC1994726 16987896

[8] Walton DS. Urrets-Zavalia syndrome following goniotomy in a child. J AAPOS. 2013;17(1):114–5. 10.1016/j.jaapos.2012.10.003 23273938

[9] Klezlova A, Liebezeit S, Prokosch-Willing V, Gericke A, Pfeiffer N, Hoffmann EM. Urrets-Zavalia syndrome after combined trabeculotomy-trabeculectomy surgery. J Glaucoma. 2018;27(4):e80–3. 10.1097/IJG.0000000000000894 29394202

[10] Tan AK, Humphry RC. The fixed dilated pupil after cataract surgery—is it related to intraocular use of hypromellose? Br J Ophthalmol. 1993;77(10):639–41. 10.1136/bjo.77.10.639 PMC504606 8218032

[11] Yuzbasioglu E, Helvacioglu F, Sencan S. Fixed, dilated pupil after phakic intraocular lens implantation. J Cataract Refract Surg. 2006;32(1):174–6. 10.1016/j.jcrs.2005.11.016 16516801

[12] Park SH, Kim SY, Kim HI, Yang SW. Urrets-Zavalia syndrome following iris-claw phakic intraocular lens implantation. J Refract Surg. 2008;24(9):959–61. 10.3928/1081597X-20081101-18 19044240

[13] Kummelil MK, Nagappa S, Shetty A, “Urrets-Zavalia syndrome after implantation of implantable collamer lens” free paper presented at ASCRS Congress, San Diego, Califonia, March 2011.

[14] Perez-Cambrodi RJ, Pinero-Llorens DP, Ruiz-Fortes JP, Blanes-Mompo FJ, Cervino-Exposito A. Fixed mydriatic pupil associated with an intraocular pressure rise as a complication of the implant of a Phakic Refractive Lens (PRL). Semin Ophthalmol. 2014;29(4):205–9. 10.3109/08820538.2013.821501 24074252

[15] Habash AA, Arfaj KA, Abdulsalam OA. Case report Urrets-Zavalia syndrome after implantable Collamer lens placement. Digit J Ophthalmol. 2015;21(3):1–11. 10.5693/djo.02.2014.09.002 PMC4902649 27330468

[16] Al-Thomali TAAA. Unilateral mid-dilated reactive pupil after same session bilateral toric Implantable Collamer Lens implantation. Saudi J Health Sci. 2016;5(1):49–52.

[17] Aralikatti AK, Tomlins PJ, Shah S. Urrets-Zavalia syndrome following intracameral C3F8 injection for acute corneal hydrops. Clin Exp Ophthalmol. 2008;36(2):198–9. 10.1111/j.1442-9071.2008.01687.x 18352891

[18] Spierer O, Lazar M. Urrets-Zavalia syndrome (fixed and dilated pupil following penetrating keratoplasty for keratoconus) and its variants. Surv Ophthalmol. 2014;59(3):304–10. 10.1016/j.survophthal.2013.12.002 24560126

[19] Huisingh C, McGwin G Jr. Cluster of Urrets-Zavalia syndrome: a sequel of toxic anterior segment syndrome. Br J Ophthalmol. 2013;97(12):1612. 10.1136/bjophthalmol-2013-303650 24081503

